# Spinal Cord Injury and Migraine Headache: A Population-Based Study

**DOI:** 10.1371/journal.pone.0135550

**Published:** 2015-08-26

**Authors:** Freda M. Warner, Jacquelyn J. Cragg, Marc G. Weisskopf, John K. Kramer

**Affiliations:** 1 School of Kinesiology, University of British Columbia, Vancouver, BC, Canada; 2 International Collaboration on Repair Discoveries (ICORD), University of British Columbia, Vancouver, BC, Canada; 3 Harvard School of Public Health Neuroepidemiology Research Group, Boston, MA, United States of America; Chi-Mei Medical Center, TAIWAN

## Abstract

Migraine headaches are a common neurological condition, negatively impacting health and quality of life. Among potential risk factors for migraine headache, risk of migraine headaches was elevated in individuals with spinal cord injury (SCI). The association between migraines and SCI is intriguing to consider from the perspective that migraine headaches may be acquired in response to damage in the spinal cord. The primary objective of this study was to further examine the association between SCI and migraine headache, controlling for potential confounding variables. A secondary objective was to determine the impact of migraine headaches on self-perceived health. Data from a sample of 61,047 participants were obtained from the cross-sectional Canadian Community Health Survey. Multivariable logistic regression was used to explore the association between SCI and migraine headache using probability weights and adjusting for confounders. The multivariable age- and sex-adjusted model revealed a strong association between SCI and migraine headache, with an adjusted odds ratio for migraine of 4.82 (95% confidence interval [3.02, 7.67]) among those with SCI compared to those without SCI. Further, individuals who experienced both SCI and migraine tended to report poorer perceived general health compared with the other groups (i.e., SCI and no migraine). In conclusion, this study established a strong association between SCI and migraine headache. Further research is needed to explore the possible mechanisms underlying this relationship. Improvements in clinical practice to minimize this issue could result in significant improvements in quality of life.

## Introduction

With an estimated lifetime prevalence of 11% in the general worldwide population, migraines are characterized by nausea, sensitivity to light and noise, and visual auras, in addition to intensely painful headaches [1]-[3] Migraine headaches are a particularly debilitating disease, negatively impacting overall health and quality of life [1], [4], [5]. Well-established risk factors for migraine headaches include family history, sex, and age [1], [2]. Specifically, migraine headaches are most prevalent in women of reproductive age [1], [2]. Somewhat more controversial, social and psychological (e.g., anxiety and mood disorders) variables have also been implicated as potential risk factors [1], [2], [4], [5]. An incidental finding of a recent population based study in Canada, increased prevalence of migraine headaches was reported among individuals with spinal cord injury (SCI) [6]. In principle, the association between migraines and SCI is intriguing to consider from the perspective that migraine headaches may be acquired in response to damage in the spinal cord, or as a function of other common SCI complications (e.g., chronic pain) [7].

As a first step towards establishing the association between migraines and SCI, the goal of the present study was to examine the prevalence of migraine headaches and SCI and the relationship between them while controlling for age and sex, as well as other potential confounders (e.g., smoking and stroke). To our knowledge, this is the first study to utilize multivariable analyses in the evaluation of migraine headaches and SCI and adjust for a variety of confounders [6], [7]. A secondary goal was to determine whether migraine headaches had a negative impact on overall health in individuals with SCI. To address our goals, the present study evaluated data from the national Canadian Community Health Survey (CCHS).

## Methods

### Data source

For this study, data were obtained from the CCHS 2010 Annual Component through the University of British Columbia. Researchers outside of pre-approved institutions may request access to data in a variety of ways detailed on the Statistics Canada website (http://www.statcan.gc.ca/eng/health/acces) The CCHS is a national, cross-sectional survey conducted by Statistics Canada [8]. The CCHS gathers data at a health region level on health-related topics including health status, health care utilization and health determinants. The target population of this survey includes individuals aged 12 years and older who reside in all provinces and territories within Canada. Excluded from this sample are persons living on reserves and Crown Lands, institutionalized individuals, full-time members of the Canadian Forces and certain remote populations. Using complex survey methods, the CCHS covers approximately 98% of their target population with a combined household- and individual-level response rate of 71.5% in 2010 [8]. Data were collected using a multistage, stratified cluster sampling technique, detailed elsewhere by Statistics Canada [8]. Data is publicly available by request from Statistics Canada, and was supplied by the University of British Columbia. Ethical approval was covered by the University of British Columbia’s Policy # 89 on Research Involving Human Subjects publicly available at http://universitycounsel.ubc.ca/files/2012/06/policy89.pdf.

### Study sample

To be included in the study sample, participants must have provided valid responses for SCI and migraine headache status, in addition to the potential confounders of age and sex. Those with invalid responses (e.g. “Not Stated”, “Refusal”, and “Don’t Know”) were excluded.

**Fig 1:**
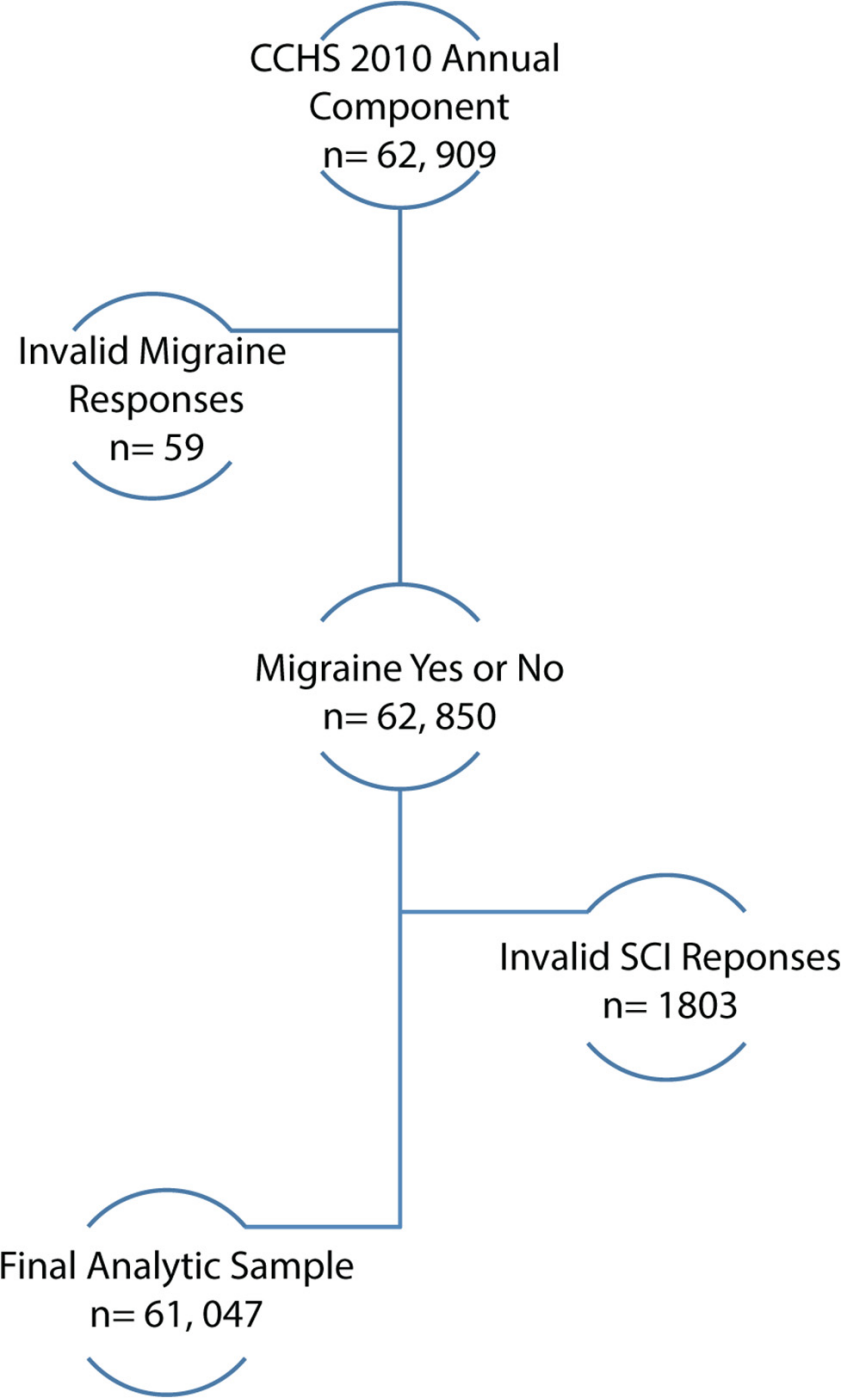
The study sample derived from the CCHS 2010 Annual Component.

Of the 62,909 respondents to the CCHS 2010 Annual Component, there was a 97.1% valid response rate for SCI status, and a 99.9% response rate for migraine headache status. All 62,909 original respondents provided valid responses for age and sex. Overall, responses that met the study’s inclusion criteria and provided valid responses for the variables above gave a final study sample size of 61,047 participants (Fig 1).

### Variable definitions

The primary explanatory variable in this study was self-reported SCI status, using the response to the CCHS question: “Do you have a neurologic condition caused by a spinal cord injury?” The outcome variable of migraine headache status was determined using the question: “Do you have migraine headaches?” Individuals were told: “Remember, we are interested in conditions diagnosed by a health professional.” Both variables are dichotomous, with possible valid responses of *Yes* or *No*. Self-report migraine headache has been validated against physician diagnosis in a prior study reporting 95% accuracy [1], [9], [10]. While there are no studies to our knowledge which examine the validity of self-reported SCI status, accurate reporting is more likely to occur for diseases with a well-defined and easily communicated diagnosis, a well as for diseases requiring hospitalization [11]. Arguably, SCI, a condition characterized by unique and distinct losses of movement and sensation, requiring hospitalization during the acute stages of injury and often in chronic stages, would satisfy these criteria [12]. As a general measure of health and quality of life, we also examined ‘self-perceived health’, obtained with the following question: “In general, would you say your health is: excellent, very good, fair, or poor. By health, we mean not only the absence of disease or injury, but also physical, mental and social-well being.”

With respect to confounding variables, we used the commonly accepted definition: a confounder is a variable that is associated with the exposure, causally associated with the outcome, and not on the causal pathway between exposure and outcome [13]. For this study, the potential confounders include age (grouped as 12–19, and then increasing in 5 year increments in order to clearly depict the non-linear effects on migraine), and sex (male or female), as these predict the primary explanatory variable (i.e., SCI) and the outcome variable (i.e., migraine). Though there are several additional risk factors for migraine mentioned above, many may fall on the causal pathway from SCI to migraine (such as depression, etc.) and therefore do not meet the criteria of a confounder [13]. An additional sensitivity analysis was however performed to observe the effect of including the additional potential confounders of heart disease, stroke, high blood pressure, smoking, and education as a proxy for socioeconomic status (all derived from self-report).

### Statistical analyses

An initial descriptive analysis provided an overview of the study sample characteristics, and bivariable logistic regression models produced unadjusted odds ratios (ORs) and corresponding 95% confidence intervals (CIs). Multivariable logistic regression was used to evaluate the outcome of migraine headache (yes/no) with the main explanatory variable of SCI while adjusting for the potential confounders of age and sex, producing adjusted ORs (AORs) and 95% CIs. Statistics Canada provides sampling weights for individuals in the study sample that are applied as probability weights to analyses for population-based estimates and appropriate estimates of variance using bootstrapping techniques (to account for uneven sampling methods) [8]. A Chi-square test was used to test for significant differences in reported self-perceived health between SCI and non-SCI populations. Fisher’s exact tests were then used within SCI and non-SCI populations to test for significant differences in self-perceived health between those experiencing migraine headaches and those without. All analyses were performed using SAS software (Version 9.4; SAS Institute, Inc, Cary, NC). Statistical significance was set at P<0.05 (i.e., where CI for ORs did not include the value one).

## Results

### Study Sample

The total study sample (n = 61,047) was equally distributed across males (49.3%) and females (50.8%; Table 1). It was approximately equally divided across age groupings, with a lower number of respondents in the younger (below 19 years of age) and older (above 60 years of age) categories.

**Table 1:** Characteristics of the Canadian Community Health Survey 2010 Annual Component Sample Examining the Relationship Between Spinal Cord Injuries and Migraine Headaches^a^.

**Variable**	**Total**	**Migraine**	
	**n (probability weighted %)**	**No (%)**	**Yes (%)**
**Study Sample**	61,047 (100)	55,091 (90.0)	5,956 (10.0)
**SCI**			
Yes	355 (0.5)	272 (0.4)	83 (1.4)
No	60,692 (99.5)	54,819 (99.6)	5,873 (98.6)
**Age**			
12–19	6,902 (11.3)	6,248 (11.4)	654 (10.5)
20–29	7,689 (16.2)	6,809 (16.2)	880 (16.0)
30–39	7,834 (14.9)	6,806 (14.3)	1,028 (19.6)
40–49	7,846 (18.1)	6,794 (17.5)	1,052 (24.3)
50–59	10,154 (17.2)	8,976 (17.2)	1,178 (17.1)
60–69	9,887 (11.8)	9,138 (12.2)	749 (8.4)
70–79	6,768 (6.9)	6,479 (7.3)	289 (2.9)
80+	3,967 (3.6)	3,841 (3.8)	126 (1.2)
**Sex**			
Male	27,658 (49.3)	25,987 (51.5)	1,671 (29.2)
Female	33,389 (50.8)	29,104 (48.5)	4,285 (70.8)
**Variable**	**Total**	**Migraine**	
	**n (probability weighted %)**	**No (%)**	**Yes (%)**
**Study Sample**	61,047 (100)	55,091 (90.0)	5,956 (10.0)
**SCI**			
Yes	355 (0.5)	272 (0.4)	83 (1.4)
No	60,692 (99.5)	54,819 (99.6)	5,873 (98.6)
**Age**			
12–19	6,902 (11.3)	6,248 (11.4)	654 (10.5)
20–29	7,689 (16.2)	6,809 (16.2)	880 (16.0)
30–39	7,834 (14.9)	6,806 (14.3)	1,028 (19.6)
40–49	7,846 (18.1)	6,794 (17.5)	1,052 (24.3)
50–59	10,154 (17.2)	8,976 (17.2)	1,178 (17.1)
60–69	9,887 (11.8)	9,138 (12.2)	749 (8.4)
70–79	6,768 (6.9)	6,479 (7.3)	289 (2.9)
80+	3,967 (3.6)	3,841 (3.8)	126 (1.2)
**Sex**			
Male	27,658 (49.3)	25,987 (51.5)	1,671 (29.2)
Female	33,389 (50.8)	29,104 (48.5)	4,285 (70.8)

^a^ All percentages are probability weighted to account for the Canadian Community Health Survey sampling design.

The prevalence of migraine headache in the final study sample was 10.0%. As predicted, migraine headache prevalence was unequally distributed among sexes, with a higher prevalence in females. Migraine headache prevalence also increased in the categories between 30–49 years of age. The prevalence of SCI was 0.49%.

### SCI and Migraine

The prevalence of migraine was higher in the population with SCI (28.9%) than in those without SCI (9.9%). Correspondingly, the unadjusted logistic regression model revealed that the odds of migraine headache was 3.69 (2.40, 5.68) times higher in those with SCI than in those without (Table 2). In the multivariable model, the adjustment for age and sex strengthened the results (Table 2): the AOR for migraine headache was 4.82 (3.02, 7.67) among those with SCI.

**Table 2:** Unadjusted and Adjusted Odds Ratios from Logistic Regression Examining the Relationship Between Migraine Headache and Spinal Cord Injuries, Canadian Community Health Survey 2010 Annual Component.

	Migraine		
Variable	Unadjusted OR (95% CI)	Adjusted OR1^a^ (95% CI)	Adjusted OR2^b^ (95% CI)
**SCI**			
Yes	3.69 (2.40, 5.68)	4.82 (3.02, 7.67)	4.43 (2.77, 7.10)
No	Reference	Reference	Reference
**Age**			
12–19	Reference	Reference	Reference
20–29	1.07 (0.91, 1.27)	1.07 (0.90, 1.27)	1.08 (0.88, 1.32)
30–39	1.48 (1.25, 1.75)	1.47 (1.24, 1.73)	1.47 (1.20, 1.81)
40–49	1.50 (1.26, 1.80)	1.48 (1.23, 1.77)	1.41 (1.14, 1.75)
50–59	1.08 (0.90, 1.29)	1.05 (0.87, 1.25)	0.91 (0.73, 1.14)
60–69	0.74 (0.62, 0.88)	0.72 (0.60, 0.86)	0.57 (0.46 0.71)
70–79	0.44 (0.35, 0.55)	0.40 (0.32, 0.50)	0.29 (0.23, 0.38)
80+	0.35 (0.26, 0.47)	0.30 (0.23, 0.40)	0.20 (0.15, 0.28)
**Sex**			
Male	Reference	Reference	Reference
Female	2.57 (2.33, 2.85)	2.67 (2.41, 2.95)	2.87 (2.59, 3.19)
**Stroke**			
Yes	1.51 (1.13, 2.03)	-	2.04 (1.43, 2.90)
No	Reference	-	Reference
**Heart Disease**			
Yes	0.98 (0.80, 1.19)	-	1.58 (1.25, 1.98)
No	Reference	-	Reference
**High Blood Pressure**			
Yes	0.96 (0.84, 1.09)	-	1.35 (1.15, 1.58)
No	Reference	-	Reference
**Smoking**			
Daily	1.52 (1.34, 1.72)	-	1.56 (1.36, 1.80)
Occasional	1.16 (0.92, 1.47)	-	1.32 (1.03, 1.68)
Former Daily	0.98 (0.86, 1.12)	-	1.21 (1.05, 1.40)
Former Occasional	1.10 (0.94, 1.29)	-	1.20 (1.02, 1.42)
Never	Reference	-	Reference
**Education**			
< Secondary	Reference	-	Reference
Secondary	1.12 (0.97, 1.31)	-	0.90 (0.76, 1.06)
Post-Secondary	1.06 (0.94, 1.19)	-	0.82 (0.72, 0.95)

^a^ Controlled for age and sex

^b^ Controlled for age, sex, smoking, high blood pressure, heart disease, stroke, and education.

### SCI, Migraine, and Perceived General Health

**Fig 2:**
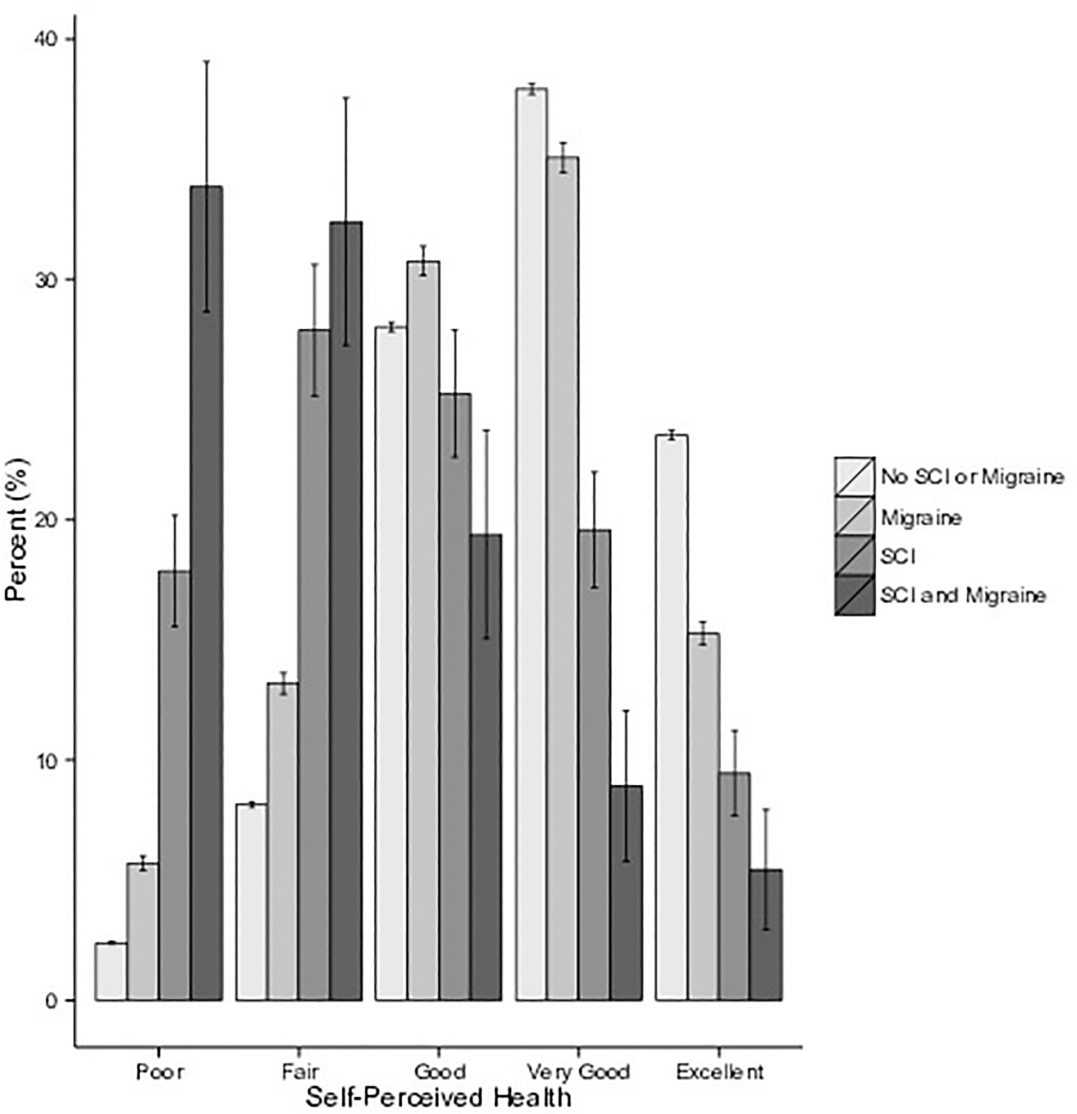
Self-perceived health in no SCI or migraine, migraine, SCI, and SCI and migraine populations. **SCI and migraine negatively affect self-perceived health more than SCI or migraine alone** ^**a**^. ^**a**^ All error bars represent the standard error of proportion.

To examine functional significance, we assessed the impact of SCI and migraine on self-perceived health. Compared to the sample without SCI, individuals with SCI reported significantly poorer self-perceived health scores (Chi-square test, p<0.001). Migraine headaches had a significant negative impact on self-perceived health in individuals with SCI (Chi-square test, p = 0.03) but not the non-SCI sample (Fisher’s exact test, p = 0.25, see Table 3). The results in Table 3 are illustrated in Fig 2.

**Table 3:** Self-reported perceived health among SCI and non-SCI populations with and without migraine^a^.

	SCI		No SCI	
Perceived health	Migraine n = 83 (%)	No migraine n = 272 (%)	Migraine n = 5864 (%)	No migraine n = 54755 (%)
**Poor**	30 (33.9)	51 (17.9)	401 (5.7)	1715 (2.4)
**Fair**	26 (32.4)	77 (27.9)	879 (13.2)	5456 (8.2)
**Good**	14 (19.4)	77 (25.2)	1863 (30.8)	16121 (28.0)
**Very Good**	9 (8.9)	56 (19.6)	1921 (35.1)	20550 (37.9)
**Excellent**	4 (5.4)	11 (9.5)	800 (15.3)	10913 (23.5)

^a^ All percentages are probability weighted to account for the Canadian Community Health Survey sampling design.

### Sensitivity Analysis

Prior to the statistical analyses, variables including smoking, high blood pressure, heart disease, stroke, smoking and socioeconomic status were excluded from the main model as it was not clear whether or not they satisfied the criteria of a confounder [13]. A sensitivity analysis was conducted in order to investigate the potential effects of these variables on the relationship between migraine headache and SCI and to test the robustness of our results. The addition of these variables in the multivariable model did not significantly affect the adjusted OR for migraine headache, as it went from 4.82 (3.02, 7.67) to 4.43 (2.77, 7.10; Table 2).

### Nonrespondents

A total of 1,862 individuals were excluded from the main analysis due to invalid answers (*don’t know*, *refusal*, or *not stated*) to the questions regarding SCI and migraine headache status. The sex of the nonrespondents (52.12% male, 47.88% female) was similar to that of the study sample (49.25% male, 50.75% female). Both the study sample and the nonrespondent group had the same median age of 40–49 years old.

## Discussion

The present study used data from a nationally representative Canadian survey to examine the relationship between SCI and migraine headache. Similar to a number of previous investigations, the odds of migraine headaches were increased in females, individuals between the ages of 30 to 50 years, individuals reporting a stroke, and smokers [1], [2]. Also in agreement with a previous report [14], migraines were associated with a 4-fold increased odds in individuals with SCI. In the present study, after adjusting for major confounding variables (e.g., age and sex), this association increased to nearly 5-fold. Interestingly, the association between migraines and SCI is drastically higher than the risk of other secondary conditions more commonly linked with SCI, including Type 2 diabetes, chronic respiratory conditions, heart disease, and stroke [15–17]. Highlighting functional significance, individuals who experienced SCI and migraines reported significantly poorer self-perceived health compared to those with SCI and no migraine.

### Spinal cord injury and migraines: Misclassifications, potential mechanisms, and impact on health

Putative mechanisms linking migraine with stroke include impaired vascular function, prominently evidenced by reduced number and function of endothelial progenitor cells [18], [19], as well as chronic inflammation [20]. In line with stroke findings, migraines could increase the risk of non-traumatic forms of SCI (e.g., spinal cord infarctions) [21]. However, the relative incidence of spinal cord infarctions is low compared to traumatic and other forms of non-traumatic SCI (e.g., tumors), and therefore alone seems to be an unlikely explanation for a relationship of such magnitude [22], [23]. Reported in approximately one third of individuals sustaining traumatic SCI [24], concomitant head injuries could also explain the association with migraines, thus representing a potential misclassification (i.e., posttraumatic headaches) [24].

Also a potential source of misclassification, SCI is well known to result in autonomic dysfunction, often characterized by a dramatic increase in blood pressure, sweating, and, occasionally, intense (non-migraine) headaches [25]. In a recent study, headaches were proposed as a key symptom underlying the detrimental effects of “blood pressure dysregulation” on health related quality of life after SCI [26]. An alternative relationship to consider is that migraine headaches are acquired more directly in response to SCI or other secondary comorbidities associated with SCI. For example, individuals with chronic pain conditions, including “spinal pain” from back and neck injuries, are at increased risk of migraines (approximately 5-fold) [27], [28]. A substantial proportion of individuals with SCI experience nociceptive and neuropathic pain [29], which in turn could increase the odds of developing migraine headaches. The overlap between neuropathic pain and migraines could be a function of similar underlying mechanisms, such as central sensitization [30], [31].

Regardless of the underlying mechanisms or potential for misclassification, an important finding of this work is that SCI and headaches have a substantial negative impact on self-perceived health after SCI. Supporting the validity of our findings, the negative impact of SCI on health status is in line with previous studies [32], [33]. Compounding the detrimental impact of SCI, individuals with migraine headaches more frequently reported “poor” self-perceived health compared to individuals with SCI without migraine (Fig 2). Although not significant, a similar pattern of poorer self-perceived health among the non-SCI population with migraines was also observed [34], [35]. Other conditions known to impact self-perceived health after SCI include the need for mechanical ventilation and shoulder pain [36], [37]. Of concern, low self-evaluations of health status are associated with higher risk of mortality, independent of the predictive contribution made by the presence of health conditions, physical disability, and life-style risk factors [35].

### Relationship with other studies in spinal cord injury

To our knowledge, three other studies have considered the prevalence of headaches after SCI. Spierings and colleagues (1992) surveyed 20 individuals with tetraplegia (18 with complete injuries), identifying common sources of headaches (e.g., bowl and bladder stimulation causing autonomic dysreflexia), but finding no evidence of migraines [38]. In a larger sample (n = 114), 15% of individuals with SCI reported migraines [39]. An Estonian study (n = 73) found that headache is the most prevalent pain condition following traumatic SCI [7]. Due to the low number of subjects and lack of comprehensive data, these studies did not control for known confounding factors that influence the prevalence of migraine (e.g., sex and age). In current study, potential confounding independent variables (i.e., associated with both the outcome and the explanatory variable) were considered. Importantly, when controlling for these variables, the odds of migraines in individuals with SCI markedly increased (Table 2), further highlighting the strength of this association.

### Future directions and Study limitations

It is important to note some of the potential limitations of the current study. First, we are utilizing self-report measures, both in term of identifying as an individual with SCI and as a migraineur. These survey variables were not validated using hospital records. While the prevalence of migraines is in line with other studies, and self-report migraine headache has been validated [1], [9], [10], some misclassification of SCI is possible. Indeed, the prevalence of SCI reported in the CCHS (0.49%) was considerably higher than that previously estimated for Canada (0.25%) [29]. Possible sources of misclassification of migraine headaches may have resulted from concomitant head trauma resulting in post-traumatic headaches, and non-migraine headaches resulting from autonomic dysfunction. Due to the limited data available, it was impossible to control for these factors [24], [25]. Importantly, despite this, misclassification would be expectedly non-differential by migraine status (i.e., occur equally in both groups).

Additionally, using CCHS data we cannot examine the impact of injury related characteristics on migraine. To better understand the relationship of migraine headaches with injury characteristics, and to determine whether the association is related to misclassification of autonomic headaches, controlling for level of injury is of paramount importance (note: autonomic headaches are only observed in individuals with injuries above T6) [25]. A more detailed clinical picture of migraine symptoms (e.g. family history and presence of aura) would also provide insight into the possibility that autonomic headaches, which are not typically associated with aura, are being misclassified [40]. Future studies should also consider the directionality of the relationship, establishing the causal pathway between SCI and migraine.

## Conclusion

In summary, we report a strong relationship between migraine headache and SCI after controlling for major confounding variables, and show that migraine headaches and SCI may compound self-perceptions of health when occurring together. To shed new light on this association and potential mechanisms, further studies are warranted.
