# Correction: Spinal Cord Injury and Migraine Headache: A Population-Based Study

**DOI:** 10.1371/journal.pone.0139181

**Published:** 2015-09-22

**Authors:** Freda M. Warner, Jacquelyn J. Cragg, Marc G. Weisskopf, John K. Kramer

There are errors in the second and third sentence of the Abstract. The second sentence should be removed completely, while the correct third sentence should read: The association between migraines and spinal cord injury (SCI) is intriguing to consider from the perspective that migraine headaches may be acquired in response to damage in the spinal cord.

There is an error in Table 1. The table was duplicated and currently presents the data twice. Table 1 should end after the first “Female” row. Please see the correct Table 1 here.

**Table 1:** Characteristics of the Canadian Community Health Survey 2010 Annual Component Sample Examining the Relationship Between Spinal Cord Injuries and Migraine Headaches^a^

Variable	Total	Migraine	
	n (probability weighted %)	No (%)	Yes (%)
**Study Sample**	61,047 (100)	55,091 (90.0)	5,956 (10.0)
**SCI**			
Yes	355 (0.5)	272 (0.4)	83 (1.4)
No	60,692 (99.5)	54,819 (99.6)	5,873 (98.6)
**Age**			
12–19	6,902 (11.3)	6,248 (11.4)	654 (10.5)
20–29	7,689 (16.2)	6,809 (16.2)	880 (16.0)
30–39	7,834 (14.9)	6,806 (14.3)	1,028 (19.6)
40–49	7,846 (18.1)	6,794 (17.5)	1,052 (24.3)
50–59	10,154 (17.2)	8,976 (17.2)	1,178 (17.1)
60–69	9,887 (11.8)	9,138 (12.2)	749 (8.4)
70–79	6,768 (6.9)	6,479 (7.3)	289 (2.9)
80+	3,967 (3.6)	3,841 (3.8)	126 (1.2)
**Sex**			
Male	27,658 (49.3)	25,987 (51.5)	1,671 (29.2)
Female	33,389 (50.8)	29,104 (48.5)	4,285 (70.8)
